# Off-label therapy targeting pathogenic inflammation in COVID-19

**DOI:** 10.1038/s41420-020-0283-2

**Published:** 2020-06-12

**Authors:** Luigina Romani, Carlo Tomino, Paolo Puccetti, Enrico Garaci

**Affiliations:** 1 Department of Experimental Medicine, University of Perugia, Perugia, Italy; 2 University San Raffaele and IRCCS San Raffaele, 00166 Rome, Italy

**Keywords:** Infectious diseases, Medical research

The world is facing a pandemic of severe acute respiratory syndrome coronavirus 2 (SARS-CoV-2) for which no proven specific therapies are available other than supportive ones. From the start of the coronavirus disease 2019 (COVID-19) outbreak, in China and in other countries patients have received off-label and compassionate use therapies, such as interferon (IFN)-α combined with the repurposed drug Kaletra, an approved cocktail of the human immunodeficiency virus (HIV) protease inhibitors ritonavir and lopinavir, chloroquine, azithromycin, favipiravir, remdesivir, steroids, and anti-interleukin (IL)-6 inhibitors, based on either their in vitro antiviral or anti-inflammatory properties.

SARS-CoV-2 is an enveloped, positive-sense, single-stranded RNA β-coronavirus similar to the severe acute respiratory syndrome (SARS-CoV) and Middle East respiratory syndrome (MERS-CoV) viruses. No clinical evidence currently supports the efficacy and safety of any drugs against coronaviruses in humans, including SARS-CoV-2. Existing antivirals and knowledge gained from the SARS and MERS outbreaks have been employed as the fastest route to fight the current coronavirus epidemic. Testing therapies approved for other indications makes senses. The World Health Organization considered remdesivir the most promising candidate to treat COVID-19, on the basis of its broad spectrum activity and clinical safety from Ebola virus disease trials. However, antivirals known to be acting at targets not playing a role in the replication of coronaviruses may fail in clinical studies.

The lack of a concurrent control group prevents any true appreciation of the beneficial versus harmful effects of the off-label use of any drugs, which might be the case for the cardiovascular effects of chloroquine/hydroxychloroquine, azithromycin, and lopinavir–ritonavir. Similarly, the adverse effects associated with the compassionate use of remdesivir could not be anticipated given the paucity of information available from previous trials. Therefore, it is appropriate to propose and test implementable hypotheses to discover new therapies for the current and any future coronavirus pandemics.

Most of the drugs in clinical trials inhibit key components of the coronavirus infection lifecycle^1^. However, the occurrence and outcome of COVID-19 infection depend on the interaction between the virus and an individual’s immune system. Many viruses multiply in the host without causing significant damage, including viruses that are capable of causing disease. However, the host response itself may lead to pathological outcomes, which may be relatively nonspecific or may result in a specific injury in target organs via cellular and humoral immune responses. Accumulating evidence suggests that some patients with severe COVID-19 infection might have a cytokine storm-like syndrome, contributing to the often lethal acute respiratory distress syndrome^2^. The severity of COVID-19 disease has been associated with increased chemokines and cytokines, such as tumor necrosis factor (TNF)-α and, to a lesser extent, IL-1β and IL-6, suggesting the occurrence of an uncontrolled inflammation in response to the virus. Of note, bats tolerate coronavirus—no inflammation in the face of an unimpaired viral load—thanks to a dampened transcriptional priming of the inflammasome sensor NLR family pyrin domain containing 3 (NLRP3)^3^, one major executor of the vertebrate inflammatory response. This suggests that targeting selective pathways of the inflammatory response—rather than interfering with the plethora of inflammatory pathways—might be a successful strategy in COVID-19 infection.

To achieve this goal, immunomodulatory agents capable of keeping the runaway inflammatory response at bay, without compromising the ability of the immune system to respond to pathogens, are urgently needed. As a matter of fact, with respect to anti-inflammatory therapy in COVID-19 infection, the use of intravenous steroids has been associated with delayed coronavirus clearance in both blood and lungs and steroids were associated with significantly increased risk of mortality and secondary infections in patients with influenza^4^. Furthermore, in spite of the documented efficacy of the IL-6 inhibitor tocilizumab in the treatment of COVID-19^5,6^, IL-6 inhibitors may cause even more profound immunosuppression than steroids, increasing the risk of sepsis, bacterial pneumonia, gastrointestinal perforation, and hepatotoxicity^7^.

Preclinical studies have pointed to the efficacy of the selective inflammatory pathways blockade in lung inflammation by drugs that are already in use in humans. Patients with COVID-19 may have features mimicking rheumatic diseases, such as arthralgias, acute interstitial pneumonia, myocarditis, leucopenia, lymphopenia, thrombocytopenia, and cytokine storm. This may suggest that drugs commonly used in rheumatology could be beneficial in COVID-19. Accordingly, several TNF-α-blocking antibodies, successfully used to treat inflammatory diseases, have been recommended for the hospitalized COVID-19 patients^8^. Similarly, IL-1β inhibitors may have significant potential at controlling hyperinflammation in severe COVID-19 disease. Among the latter, anakinra is the recombinant form of the IL-1 receptor antagonist, known to inhibit NLRP3 via blockade of the IL-1 receptor I. Anakinra is currently used to treat a wide range of diseases that goes beyond its approved indications for rheumatoid arthritis and cryopyrin-associated periodic syndromes, to encompass cancer and chronic inflammatory diseases^9^. Anakinra potently inhibited pathogenic NLRP3 activation in the murine lung and human bronchial epithelial cells from patients with lung infection and inflammation, and it concurrently inhibited IL-1β, TNF-α, and IL-6 production^10^. Compared to other biologics, anakinra has an unparalleled safety profile. It is currently being explored in a phase IIa, randomized, placebo-controlled, double-blind, cross-over study in patients with cystic fibrosis (NCT03925194). Thus patients with severe COVID-19 may likely benefit from therapeutic options that include, among others, NLRP3 antagonists and IL-1 inhibitors to inhibit unwanted inflammation while preserving antimicrobial defense. Currently, anakinra is being trialed in a randomized placebo-controlled study in children and adults with COVID-19-associated cytokine storm syndrome in China (NCT02780583) and in a phase 2/3, randomized, open-label, multicenter study investigating the efficacy and safety of intravenous administrations of anakinra in Italy (Sobi.IMMUNO-101, March 20, 2020). AIFA has also recently approved the compassionate use of canakinumab, a fully human monoclonal antibody that neutralizes the bioactivity of human IL-1β, in patients with COVID-19 (April 10, 2020).

Consistent with the observation that a robust cytotoxic T lymphocyte response plays a vital role in clearing coronavirus, lymphopenia, affecting both CD4+ and CD8+ T lymphocytes, has been described in patients with both SARS-CoV and MERS-CoV infections and correlated with the severity of the disease^11,12^. For its ability to stimulate innate and adaptive immune responses, thymosin alpha1 (Tα1), a naturally occurring thymic peptide of 28 amino acids, is used worldwide as an immunomodulator in a wide range of clinical indications^13,14^ (Fig. 1). Owing to its ability to promote IFN type I production and activation of CD8+ T cells via stimulation of innate receptors, Tα1 has been registered as a treatment or prevention of respiratory viral infections and as an immune adjuvant in influenza vaccination of the elderly and hemodialysis patients^13,14^. Either in monotherapy or in combination with IFN-α, Tα1 has been approved in 30 countries for treatment of chronic viral infections, including chronic hepatitis, chronic hepatitis C, and HIV^13,14^. During the 2009 pandemic outbreak of H1N1 influenza, Tα1 provided an earlier and greater response to the vaccine in a clinical study with Focetria™ MF59-adjuvanted monovalent H1N1 vaccine^15^. Interestingly, the use of Tα1 has also been claimed in SARS^16,17^. By reducing the mortality rate and decreasing the incidence of secondary infections, Tα1 has shown a promising activity also in sepsis^18^. Not surprisingly, Tα1 has been included in a recent randomized, open, controlled trial in combination with darunavir/cobicistat or lopinavir/ritonavir in the treatment of COVID-19 (registration number ChiCTR2000029541)^1^. Owing to its ability to maintain immune homeostasis by activating the tolerogenic pathway of tryptophan catabolism via the immunoregulatory enzyme indoleamine 2,3-dioxygenase 1, Tα1 specifically potentiated immune tolerance in diseased lungs, breaking the vicious circle linking chronic lung inflammation and infections^19^ and restoring ciliary beating function^20^. Thus Tα1 holds promise for representing an ideal candidate drug that, as suggested^21^, will boost immunity at an early stage of SARS-CoV-2 infection and promote immune tolerance and tissue homeostasis in the severe respiratory stage.

**Fig. 1:**
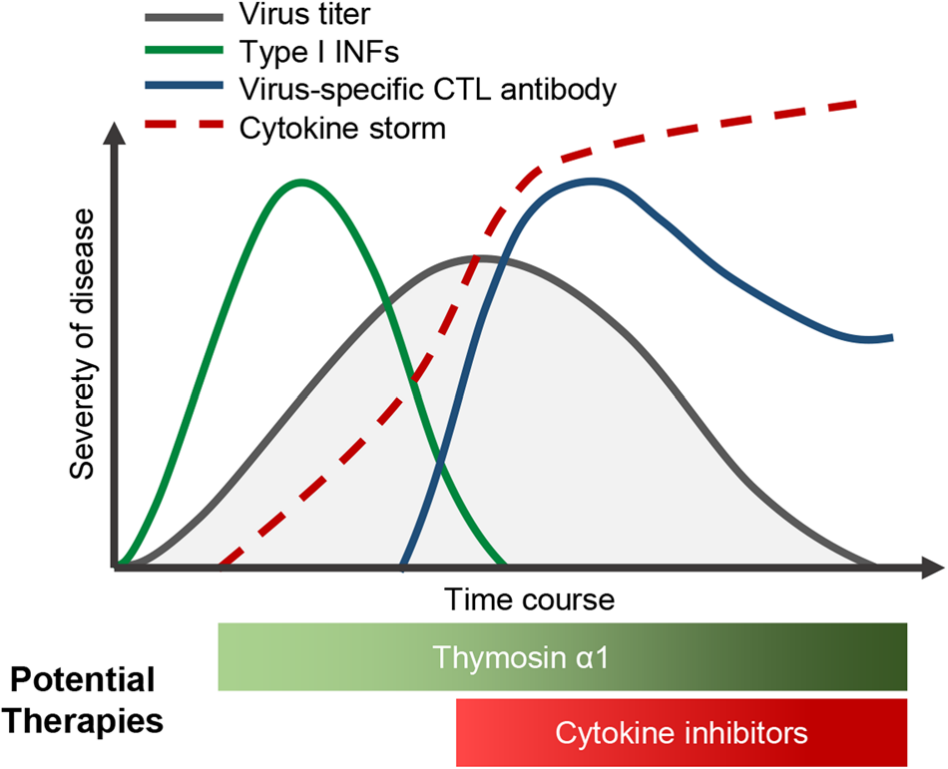
The figure illustrates the dynamics of the antiviral innate and adaptive immune response and its dysregulation potentially leading to the progression of COVID-19 infection. As explained in the text, by virtue of its multifaceted activity, thymosin α1 may boost immunity at an early and late stages of SARS-CoV-2 infection and, like cytokine-specific antagonists, may adversely affect the cytokine storm by promoting immune tolerance and tissue homeostasis in the severe respiratory stage.

Even though virtual screening makes it possible to discover molecules relatively quickly, these compounds still need to be experimentally tested before clinical use. Drug repurposing, with a breadth of a dual activity against the virus and the host, may thus rightly come into play with safety and low cost.

## Note added in proof

After the submission of the present comment, the following papers (shown in References) have been published:

1. Pontali, E. et al. Safety and efficacy of early high-dose IV anakinra in severe COVID-19 lung disease. *J. Allergy Clin. Immunol*. **S0091–6749**, 30634–30635 (2020). 10.1016/j.jaci.2020.05.002.

2. Filocamo, G. et al. Use of anakinra in severe COVID-19: a case report. *Int. J. Infect. Dis*. **S1201–9712**, 30333–30337 (2020). 10.1016/j.ijid.2020.05.026.

3. Day, J. W., Fox, T. A., Halsey, R., Carpenter, B., & Kottaridis, P. D. Br J. IL-1 blockade with anakinra in acute leukaemia patients with severe COVID-19 pneumonia appears safe and may result in clinical improvement. *Haematology*. 10.1111/bjh.16873 (2020).

4. Dimopoulos, G. et al. Favorable anakinra responses in severe COVID-19 patients with secondary hemophagocytic lymphohistiocytosis. *Cell Host Microbe*. 10.1016/j.chom.2020.05.007 (2020).

5. Liu, Y. et al. Thymosin Alpha 1 (Tα1) reduces the mortality of severe COVID-19 by restoration of lymphocytopenia and reversion of exhausted T cells. *Clin. Infect Dis*. 10.1093/cid/ciaa630 (2020).
